# A conversation with Olufunmilayo Olopade

**DOI:** 10.1172/JCI178495

**Published:** 2024-01-16

**Authors:** Ushma S. Neill

Physician and geneticist Dr. Funmi Olopade is the founding director of the Center for Cancer Genetics and Global Health at the University of Chicago (Figure 1). Olopade’s research is focused on gaining a better understanding of the root causes and genomic basis of cancer in diverse populations. She is internationally renowned for her work in inherited cancer syndromes and for her clinical expertise in early detection and prevention of breast cancer in high-risk women. To hear her talk about doing physics problems for fun, see the full video on the *JCI* website at www.jci.org/videos/cgms

*JCI:* What were you like as a child?

Olopade: I grew up in Nigeria. I started school in a very small town called Ijebu Igbo, although I was born in Abeokuta. I’m number five of six children and was very quiet as a child. My father was a pastor. In his days, there were only three professions you could consider: a teacher, a pastor, or a doctor. He had the opportunity to go to school to be a teacher, but then he got into the missionary society and became a pastor.

Being in a missionary family meant we grew up in very small towns and the focus was to get people educated and thinking about the world larger than themselves. Most of my early days were spent reading books and magazines and newspapers. We would pour over *Time* magazine every week when it got delivered like it was the best thing.

Like many who grew up in a small town in Nigeria, you have to leave home to get the best education, so I went to boarding school when I was 12. By the time I was in sixth form, there were only 18 of us in the advanced science class in my all-girls school. We had wonderful teachers, and we tracked in either arts or science early on. I was quite strong in mathematics and physics. My brother had already become an engineer, and my father really wanted a doctor in the family, so after my O-levels, which is really where you did more science, I went to Queen’s College in Lagos. That’s where for A-levels, I did physics and dropped math for biology. I did not like my biology classes as much as physics, but still excelled to get to medical school.

*JCI:* Did you have any early clinical inclinations, like toward cardiology or anesthesiology or surgery?

Olopade: I enjoyed medicine and pathology the best. And Robbins’ *Textbook of Pathology* was so fun to read, and I had this habit of reading every book in medical school from cover to cover, and people thought that was nerdy, but I enjoyed it.

Physiology was great and biochemistry too. Anatomy felt like a lot of tedious memorizations, but once we finished the preclinical part of medicine and got to the clinical years, it was so wonderful to be a medical student and to learn that we didn’t have all the answers. My pediatric rotation was fascinating because we had an American-trained pediatrician, and in medicine, we had a diabetologist who practiced the American way, which was very different from the British way. I began really thinking about whether any of those specialties would interest me.

I left Nigeria still eliminating things that I didn’t like. Pathology, I would’ve done, except I didn’t like morbid anatomy; I couldn’t imagine having to do autopsies. In coming to America, I wanted to do internal medicine and I had role models who were in cardiology. We had done one heart transplant during my rotating internship in Nigeria. He was a kid with rheumatic heart disease, and it was the first cardiac transplant in Nigeria. I was on that service; I worked hard but unfortunately the patient died and I kept thinking it was a futile exercise. If the child had had penicillin, he would never have damaged his heart — that got me thinking about prevention. Those were moments that were transformative for me in thinking about how I wanted to spend the rest of my life as a doctor.

*JCI:* How did you happen to move to Chicago for your medical residency?

Olopade: One of my habits in medical school was to wake up to the radio program *Voice of America*. During the Iran hostage crisis, I loved Jimmy Carter and I loved everything that was happening in America. Given the profits from finding oil in Nigeria, we all went to medical school for free, and the government gave us some money to invest in our education that allowed us to discover the world. My brother was already in graduate school at Stanford, and so during the summer I decided to use my bursary to travel and visit him. I loved it. I thought everything was big in America.

*JCI:* Well, that is often true. How did you pick Chicago and the program at Cook County for your residency?

Olopade: I tried to interview at different places. Since my brother was at Stanford, I tried to get interviews at San Francisco General and Stanford. But those programs didn’t take international graduates. I got advice to start at a public hospital. We had a friend in Chicago, and he was willing to welcome us.

In parallel, at that time we didn’t know what caused AIDS, but people were dying, and we didn’t know why they were dying; the public hospitals were short staffed. The minute I walked into Cook County Hospital in Chicago, they offered me a job. It was like — any warm body standing just come on in.

*JCI:* You moved on to do a clinical fellowship at the University of Chicago?

Olopade: I was looking to do research. That was hard at Cook County, as we were so busy and so understaffed. I ended up doing another year of chief residency and during that year, I had time to reflect on what I wanted to do. There was so much cancer in the community, and the patients suffered so much. But those who came from no matter what part of the city got better. I didn’t see that in Nigeria; everyone in Nigeria died, and there was so much stigma around cancer.

During my chief residency I used autopsies to learn. I took it upon myself to consent families of nearly every patient who died on our services to do autopsies. These were mostly Black patients in Chicago, and during that year, we got our autopsy rate to almost 70%. We learned a lot and got better at taking care of very sick patients. Everyone I asked said, “Sure.” I thought, if I could go to a place where I can do research, this will be phenomenal because then people are not going to die from cancer because we will figure it out one day. I applied to University of Chicago and embraced immersing myself in research.

*JCI:* You joined the lab of the legendary leukemia geneticist Janet Rowley. Was this the dawn of your interest in genetics?

Olopade: Nobody goes to University of Chicago without learning about Janet Rowley! But she was honestly very approachable. You only needed to be in a room with her once to want to be like her. I was lucky that she was welcoming and supported late bloomers like me. By this time, I had three children; my colleagues in the fellows’ room all were very good at babysitting my children while I finished an experiment in the lab.

Dr. Rowley was very adamant I should do molecular biology. So I knew molecular biology, and she knew cytogenetics. We worked together to curate chromosome aberrations in solid tumors. I realized that we didn’t really have much on solid tumors. At the time, you couldn’t grow them in culture, there was no such thing as organoids, and we didn’t know nearly enough about them. The few cell lines that were available for solid tumors were only from melanoma, head and neck cancers, and lung cancers. I studied all of those cancers except leukemia because I knew I couldn’t compete with Dr. Rowley and all the other great hematologists at the University of Chicago. That turned out to be really a good strategy for me.

*JCI:* Was your perspective that genetics played a larger role in breast cancer in the African diaspora (more than poverty or medical neglect) considered heretical or revolutionary at the time? How did you go about building the coalitions and data to support it?

Olopade: I happened to have gone to a Gordon Conference and run into Francis Collins and Mary-Claire King, who were mapping genes on different chromosomes, but using human families as their model. This inspired me and gave me an idea. While I was looking for a tumor-suppressor gene on chromosome 9 — I was going to knock it out and have an animal model to do functional studies — Mary-Claire and I both got scooped. I didn’t get the *p16* gene on chromosome 9 even though I had mapped all that region, and she didn’t get *BRCA1*. And misery loves company, right? But it also allowed me to play to my strengths — in the clinic.

My patients became the laboratory for me, as we were looking for melanoma families to map the gene on chromosome 9. But I didn’t have enough melanoma families; on the contrary, the same year *BRCA1* was identified, and I realized I had a boatload of breast cancer families. As soon as the race was on to find tumor-suppressor genes, patients were calling and coming to my clinic from south, north, everywhere. When we started the Cancer Risk Clinic, I was still racing to find the gene on chromosome 9, but families with breast cancer were coming in large numbers.

I had a startup package that included a genetic counselor and a nurse. The University of Chicago embraced the idea that I could create something unique and different from Janet so that my scholarship couldn’t be attributed to her. I was able to move the needle for *BRCA1* research because the first few families that helped nail *BRCA1* were Black families, including families with five generations of slavery in their family history. I was single-minded about tracking this. I went to family reunions down south. The patients trusted me, while also wanting to be part of the solution. I was furthermore fortunate to have an opportunity to apply for the Department of Defense Idea Award, which asked for anyone who had ideas on what to do about breast cancer to write a proposal. We had published our first paper on breast cancer in extended African-American families. They didn’t have what at that time everyone was thinking were “Jewish mutations” — they just had heterogeneous mutations/variants of unknown significance. The minute you see variants of unknown significance, you have to continue to dig deeper. We were able to link up with those who were studying breast cancer, like Charles Perou, who had looked at basal-like breast cancer.

We got *BRCA1* in ‘94; *P16* was 1994 also. My husband and I had gone back to our Nigerian medical school in 1996 as alumni speakers, and we were trying to tell our colleagues and the medical school students about the great discovery of *BRCA1*. Everybody was like, what? This is highfalutin; it doesn’t apply to us. I had left in 1983 and they were still teaching students the common things: if you see a mass, it’s tuberculosis until proven otherwise. I started off thinking that it was my obligation to teach them about the future of medicine; remember, my father being a missionary had taught me that knowledge is power and that ignorance kills. I wanted to give back to Nigeria and show them the data and convince them to join the effort.

In 2004, we had the first international workshop on breast and cervical cancer. That’s eight years from my encounter in 1996. By the end of that meeting in Lagos, we had a ten-point communique: my Nigerian colleagues said, “We want immunohistochemistry, we want to study breast cancer, we want to study cervical cancer, we want to do genetics research.” They put in terms of how we could continue the collaboration. That ten-point communique is what I’ve used to think about what I study and how I bring my colleagues in Nigeria along so that we keep pushing the science forward.

*JCI:* If you could not have been a physician or a scientist, what other profession do you think would’ve kept you as engaged?

Olopade: I love debates, and I also love acting. I wanted to be a lawyer because law as a career would really allow me to be able to debate. But then the science and the math could always get me focused. At one point, I thought I’d go to divinity school, after I’d finished with medicine. But then I realized I believed too much in science and technology to go to divinity school. I love being a doctor, and I’ve been fortunate that my patients trust me, and the fact that I don’t know nearly enough to help every patient just keeps me going.

## Figures and Tables

**Figure 1:**
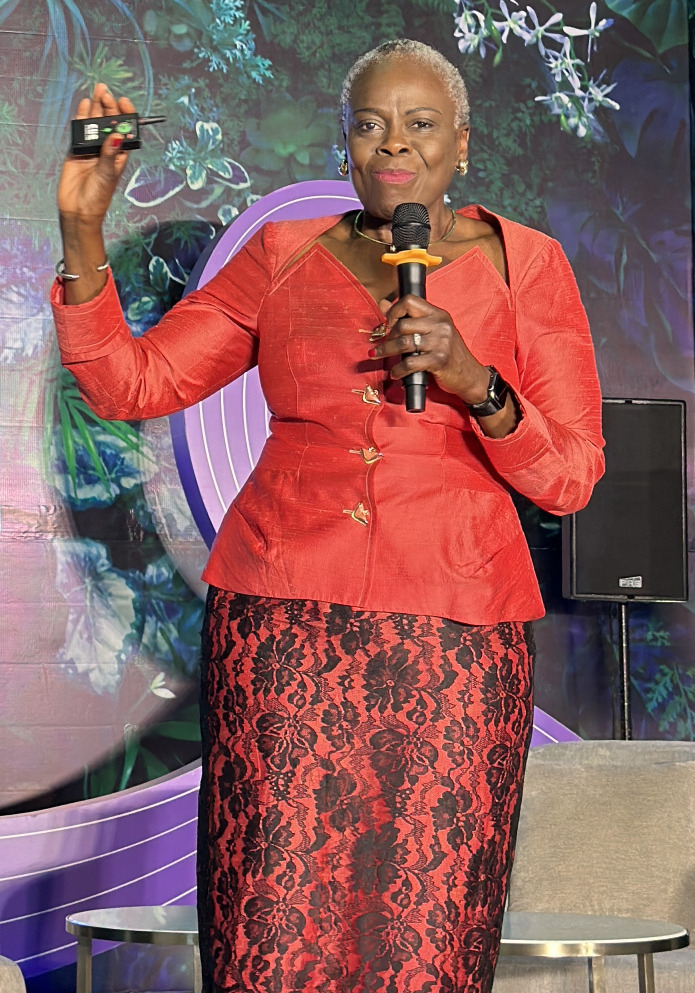
Olufunmilayo Olopade speaking at a conference in Vietnam in August 2023.

## Version 1 01/16/2024

Electronic publication

